# Acoustic Features of Pain-Induced Vocalisations Are Associated with Subsequent Growth and Temperament Following Thermal Disbudding in Goat Kids: A Preliminary Study

**DOI:** 10.3390/ani16162530

**Published:** 2026-08-13

**Authors:** Zhengyu Fu, Yiming Gong, Wenyue Hou, Li Li, Hongping Zhang

**Affiliations:** Farm Animal Genetic Resources Exploration and Innovation Key Laboratory of Sichuan Province, College of Animal Science and Technology, Sichuan Agricultural University, Chengdu 611130, China; zhengyu-fu@foxmail.com (Z.F.); g18080010835@163.com (Y.G.); houwenyue516@163.com (W.H.)

**Keywords:** goat kids, disbudding, vocalisation, acoustic analysis, MFCCs, vocal phenotype

## Abstract

Whether vocalisations during disbudding predict later outcomes in goat kids is unknown. In this preliminary study, we allocated 18 female Tianfu goat kids to disbudding (*n* = 9) or sham (*n* = 9). Disbudding used a hot iron without analgesia; sham kids received an unheated iron. Vocalisations, tail flicks, and struggles were recorded. Fundamental frequency (F0) and Mel-frequency cepstral coefficients (MFCCs) were extracted. Body weight and temperament were assessed at 90 days post-procedure; home-pen behaviour was scan-sampled at 60 and 90 days post-procedure. Two kids were excluded due to insufficient recordings, leaving 16 (9 disbudded, 7 sham). Disbudded kids vocalised more frequently (14.5 vs. 8.1 calls/min, *p* = 0.0007) and had lower minimum F0 (283.4 vs. 366.1 Hz, *p* = 0.020), with significant MFCC shifts. Several acoustic features correlated with body weight at 90 days post-procedure (mfcc_12_std: *r* = 0.903, *p* = 0.001) and temperament (mfcc_2_mean: *r* = −0.788, *p* = 0.012) after controlling for disbudding weight. The first principal component of acoustic features (PC1) correlated with lower body weight at 90 days post-procedure in disbudded kids (*r* = −0.786, *p* = 0.036), but not in shams (*r* = −0.257, *p* = 0.623). No subgroup differences were found in home-pen behaviours. These findings indicate that acute pain vocalisations may reflect individual stress reactivity—a possible ‘vocal phenotype’—and, with validation, could inform non-invasive screening for at-risk kids.

## Simple Summary

This study investigated whether the vocalisations that goat kids make during disbudding could provide information about their later growth and temperament. We recorded calls from kids undergoing actual disbudding or a sham procedure, and measured their body weight and reactivity three months later. Kids that experienced real disbudding called more often and produced calls with distinct acoustic features compared to those in the sham group. Certain acoustic features were linked to lower weight and more reactive temperament at three months of age, but only in kids that underwent the painful procedure. These findings indicate that vocal responses to pain may reflect individual differences in stress sensitivity that influence later development, and that recording vocalisations could offer a non-invasive way to identify kids needing additional care after painful procedures.

## 1. Introduction

Thermal disbudding of goat kids is a routine husbandry practice globally to prevent horn-related injuries to farm personnel, conspecifics, and the animals themselves, as well as to facilitate herd management. However, the procedure causes marked tissue damage and raises substantial animal welfare concerns. Although several analgesic approaches have been evaluated, disbudding is still commonly performed without effective pain relief in commercial settings [1,2,3]. Current pain mitigation strategies, including cornual nerve blocks, topical anaesthetics, and systemic nonsteroidal anti-inflammatory drugs (NSAIDs), have not consistently eliminated physiological and behavioural pain responses to cautery disbudding in goat kids [4,5], underscoring the need for improved welfare interventions and more sensitive tools to quantify pain severity.

Current pain assessment in disbudded goat kids relies primarily on procedure-time behavioural counts, including vocalisations, struggling, and tail flicking, and on physiological measures such as plasma cortisol [1,2], serum haptoglobin and non-invasive infrared thermography of the horn bud region [6], each of which has notable limitations. Behavioural counts primarily capture the quantity rather than the qualitative intensity of the pain response. For instance, struggling is heavily influenced by restraint and handling per se, while tail flicks have shown considerable variability across studies [3,7]. While physiological measures offer greater objectivity, they also present specific drawbacks. Cortisol and haptoglobin assessments require invasive blood sampling, which can induce additional stress and potentially confound the measured response [7]. Conversely, although infrared thermography is non-invasive and has been applied in goat disbudding studies [6], it requires specialised equipment and careful standardisation of environmental conditions. Consequently, there remains a need for non-invasive indicators that can capture both the occurrence and magnitude of pain-related responses under practical farm conditions.

Bioacoustic analysis offers a non-invasive alternative that has been validated across farm species. In piglets, surgical castration alters call duration, peak frequency, and spectral entropy, while the number of calls does not [8]. In beef cattle, hot-iron branding increased the fundamental frequency range and peak frequency of vocalisations [9]. Recent work in goats has further demonstrated that Mel-frequency cepstral coefficients (MFCCs) are the dominant acoustic features for classifying vocalisations across multiple welfare states, particularly for identifying extreme physical distress [10]. However, to our knowledge, no study has systematically characterised the acoustic structural changes (e.g., MFCCs and other spectral–temporal features) in vocalisations of goat kids specifically during disbudding, representing a key gap in species-specific pain assessment.

Beyond acute pain, there is growing evidence that early-life painful procedures can shape long-term phenotypic outcomes. Rodent models show that neonatal inflammatory insults induce persistent changes in pain sensitivity, cognition, and reproductive traits, with some transgenerational effects also observed in sheep [11,12]. In calves, disbudding can lead to chronic trigeminal sensitisation, persisting up to 105 days post-procedure [13]. In goat kids specifically, while pain mitigation during disbudding has been shown to improve acute responses, its effects on longer-term health and performance remain poorly understood [14]. Even less is known about whether individual differences in acute pain responses are associated with later developmental outcomes such as body weight and temperament at 90 days post-procedure. This absence of early prognostic markers limits the ability to target pain mitigation to those individuals most at risk of negative long-term effects.

Vocal individuality has been demonstrated across ungulate species. In goat kids, mothers can recognise their own offspring’s bleats as early as 2 days postpartum, and sonographic analysis reveals significant interindividual differences in call duration, fundamental frequency, and harmonic structure [15]. Machine learning approaches further confirm that goat kid contact calls carry cues to individual identity, group membership, and age, with artificial neural networks achieving 71% accuracy for individual classification [16]. However, emotional contexts modulate the expression of vocal individuality: across seven ungulate species, calls produced in negative-valence contexts contain less individual information than those in positive-valence contexts, although each species retains at least one acoustic parameter that reliably signals individuality across emotional states [17]. It remains unknown whether acute surgical pain—a negative-valence context of exceptionally high arousal—overrides or amplifies pre-existing individual differences in vocal structure. This raises a critical question: during an acutely painful procedure such as disbudding, do goat kids’ vocalisations retain sufficient individual information to serve as a marker of differential vulnerability to long-term developmental outcomes?

To address these gaps, the present study therefore aimed to: (1) quantify the effects of thermal disbudding without analgesia on both vocalisation rate and acoustic structure (including fundamental frequency and MFCC features) in goat kids; (2) test whether acoustic features recorded during the acute pain event are independently associated with body weight and temperament at 90 days post-procedure, controlling for body weight at disbudding; and (3) explore the feasibility of vocal responses as a non-invasive early marker of long-term welfare outcomes. To reflect real-world management constraints, we included only female kids, as male kids are rarely disbudded in commercial practice and horn size has been linked to semen quality in breeding bucks [18]. We hypothesise that if vocal structure during pain carries information about individual stress reactivity (a ‘vocal phenotype’), then acoustic features may be associated with later growth and temperament, but only in animals that experienced the painful procedure.

## 2. Materials and Methods

### 2.1. Animals, Husbandry, and Management

The experiment was conducted at the Tianfu Goat Breeding Farm of Sichuan Agricultural University (Ya’an, China) from October 2025 to February 2026. Eighteen newborn female Tianfu goat kids from the same farm were enrolled. Kids were assigned to a disbudded group (*n* = 9) and a sham group (*n* = 9). To control for dam and litter effects, kids from the same litter were split across the two groups. For twin litters, one kid from each pair was randomly allocated to each group. For triplet litters, two kids were randomly assigned to one group and the remaining kid to the other, with the group receiving two kids determined at random.

At birth, body weights did not differ between groups (disbudded: 3.18 ± 1.03 kg; sham: 3.15 ± 0.74 kg). Disbudding and sham procedures were performed when kids were 11.2 ± 3.0 days of age. Body weight on the day of the procedure also did not differ between groups (disbudded: 5.60 ± 0.87 kg; sham: 5.68 ± 0.91 kg). Due to insufficient call recordings and incomplete video coverage, two sham kids were excluded from behavioural and acoustic analyses, yielding a final sample of 16 kids (9 disbudded, 7 sham) for all procedure-related analyses.

At the time of disbudding, kids already had free access to solid feed and water, and received supplementary milk replacer 3–6 times daily depending on their growth stage. Weaning was carried out gradually: from 55 days of age, the daily separation time between kids and dams was progressively increased, and weaning was completed by approximately 74 days of age. After gradually weaning, dams were removed, and male and female kids were separated. The female kids from both the disbudded and sham groups were then combined into a single pen (6.35 m × 6.5 m) for the remainder of the experiment. The pen contained two freely connected areas: a feeding area with plastic slatted flooring and feed troughs (2.85 m × 6.5 m), and a resting area with concrete flooring, an electric heating plate, and heating lamps (3.65 m × 6.5 m). All procedures were approved by the Sichuan Agricultural University Animal Ethical and Welfare Committee (approval No. 20251005) and were conducted in accordance with the animal welfare guidelines established by this committee.

### 2.2. Disbudding and Sham Procedure

Disbudding was performed using thermal cautery with an electric cautery iron (220 V, 500 W). The cautery iron was preheated until the tip glowed red (approximately 15 min). All procedures were carried out by the same experienced veterinarian. Each kid was placed on the operator’s lap in a ventrally suspended posture, with an assistant holding the kid’s four legs. The operator restrained the kid’s head with one hand and applied the heated cautery iron to each horn bud with the other. The procedure was considered complete when the horn bud corium appeared fully cauterised; the total restraint time from the start of the procedure until the kid was released averaged 390 ± 115 s, comparable to values reported in [2]. No analgesic, sedative, or local anaesthetic was administered, in accordance with common commercial farm practice in the region. The sham procedure was identical in every respect, except that an unheated cautery iron was used. To standardise handling time across groups, the sham procedure was held for the mean duration of the disbudding procedure (390 s), with the unheated cautery iron applied to each horn bud using the same pressure and sequence as in the disbudded group. The time taken to return the kid to its home pen was also kept consistent between groups.

### 2.3. Behavioural Observations During Disbudding

The entire disbudding or sham procedure was video-recorded. Behaviour was later scored by a single observer using BORIS (v. 9.9) software [19] from the moment the kid was placed in restraint until 5 s after the last horn bud was treated. Three behaviours were quantified according to the definitions and recording methods detailed in Table 1. Frequencies were calculated as counts per minute of the total observation period.

### 2.4. Acoustic Recording and Feature Extraction

Audio was extracted from the same video recordings. The video camera recorded audio in WAV format at a sampling rate of 48 kHz with 16-bit depth and a constant bitrate of 1536 kbps. The camera was mounted on a tripod at a fixed distance from the kid, ensuring consistent recording conditions.

A trained experimenter then screened all excerpts and retained only high-quality calls, excluding those with signal-to-noise ratio <20 dB, those with waveform clipping, or those contaminated by handling noise. After screening, the number of retained calls per kid ranged from 6 to 19 (mean ± SD: 10.1 ± 3.4), with sham kids retaining 6–13 calls and disbudded kids retaining 7–19 calls. Acoustic analyses were performed in Python (v. 3.14.2) using the librosa library (v. 0.11.1) [21]. For each call, the following features were extracted.

Mel-Frequency Cepstral Coefficients (MFCCs): The first 13 coefficients (mfcc_0 to mfcc_12) were computed with an FFT window of 2048 samples, a hop length of 512 samples, and 128 Mel-filter banks. For each coefficient, the mean and standard deviation across all frames of the call were calculated, yielding 26 MFCC variables per call (mfcc_0_mean, mfcc_0_std, …, mfcc_12_mean, mfcc_12_std) [22]. No liftering was applied.

Fundamental Frequency (F0): F0 was tracked using the probabilistic YIN algorithm (pYIN) implemented in librosa.pyin [23]. Frames classified as unvoiced by the pYIN model were treated as missing. From the voiced frames of each call, the following statistics were computed: mean (f0_mean), standard deviation (f0_std), minimum (f0_min), maximum (f0_max), range (f0_range), and median (f0_median).

Loudness: Frame-level root-mean-square (RMS) energy was calculated with librosa.feature.rms. From the frame-level RMS values of each call, the mean (rms_mean), standard deviation (rms_std), and maximum (rms_max) were recorded.

For each kid, the above call-level features were averaged across all of the kid’s calls to obtain a single set of acoustic variables for statistical analysis. Representative spectrograms from each of the four subgroups defined by treatment and PC1 score (Disbudded-H, Disbudded-L, Sham-H, Sham-L) were generated (librosa.stft) and displayed with librosa.display.specshow (librosa v. 0.11.1).

### 2.5. Body Weight and Temperament Assessment

Body weights were measured on the day of disbudding (0 d) and again at 60 and 90 days of age. Temperament was scored during the 90-day weighing using a 1–5 scale [24], with higher scores indicating greater reactivity (Table 2). The same experienced handler assigned the score while the scale reading stabilised.

### 2.6. Scan-Sampling of Behaviour at 60 and 90 Days After the Procedure

At 60 and 90 days after the procedure, behaviour in the home pen was video-recorded continuously for 48 h (two consecutive 24 h periods). From these videos, still images were automatically extracted every 30 min using a custom Python (v. 3.14.2) script. Each kid in each image was later coded into one of three mutually exclusive behavioural states (Table 3). When a kid’s identity was ambiguous from the image alone, the corresponding video footage was reviewed. All kids were individually recognisable by their unique coat colour patterns.

For each observation day, the number of scans in each behavioural state was counted for each kid and expressed as a proportion of that kid’s total scans on that day. Data from the two observation days were then averaged, yielding a single value per behavioural state for each kid at 60 days and at 90 days after the procedure.

### 2.7. Statistical Analysis

Statistical analyses were conducted in Python (v. 3.14.2) using libraries including scipy.stats, numpy, and pingouin. Sample size was determined a priori using a power analysis based on effect sizes estimated from previous studies on pain-related outcomes in disbudded goat kids [2,5,25]. With α = 0.05, power = 0.8, and a conservative effect size of Cohen‘s *d* = 1.5, the required sample size per group was calculated as *n* = 8.06 using the pwr package in R (v. 4.4.3). Accordingly, we set the sample size at *n* = 9 per group. Before each group comparison, normality was tested with the Shapiro–Wilk test and homogeneity of variance with Levene’s test. Mean differences were then assessed as follows: if both groups met normality and equal variance assumptions, Student’s *t*-test was used; if normal but with unequal variances, Welch’s *t*-test was used; if normality was violated, the Mann–Whitney *U* test was used. For comparisons between treatments over time (e.g., body weight), separate tests were performed at each time point rather than using a repeated-measures ANOVA, as the data at some time points did not meet the normality assumptions required for repeated-measures analysis. Effect sizes were reported as Cohen’s *d* for *t*-tests and *r* = *Z*/√*N* (where N is the total sample size of the two groups combined) for Mann–Whitney *U* tests. All correlations were assessed using Spearman’s rank-order correlation, as the data were non-normally distributed.

Kids were classified into subgroups with higher (H) and lower (L) PC1 scores based on their acoustic profiles. Of the 16 kids with acoustic data from the procedure, three (two disbudded, one sham) were moved to other pens during post-weaning regrouping and could not be tracked by the camera system. The remaining 13 kids were used for PCA-based classification. All extracted acoustic features (MFCCs, F0, and RMS variables) were first standardised (*z*-scores). Principal component analysis (PCA) was performed on the standardised features; the first principal component score was extracted as a composite acoustic response score. Within each group (disbudded and sham) separately, kids were divided at the median of this PCA score: those above the median were labelled high-PC1 (H), and those below the median low-PC1 (L). This yielded 7 H kids (4 disbudded, 3 sham) and 6 L kids (3 disbudded, 3 sham). At the 90-day observation, one L sham kid escaped from the pen and no behavioural data could be recorded; this kid was therefore excluded from the 90-day scan-sampling analysis only.

Group comparisons of behaviour frequencies and acoustic features were performed between the disbudded and sham groups. Partial Spearman correlations were used to examine associations between acoustic features recorded during the procedure and body dimensions and temperament at 90 days after the procedure within the disbudded group only, controlling for body weight on the day of disbudding. For scan-sampled behaviours at 60 and 90 days after the procedure, pairwise comparisons were conducted among the four subgroups defined by treatment and PC1 score (Disbudded-H, Disbudded-L, Sham-H, Sham-L) to examine whether the overall acoustic response was associated with later behaviour. All statistical tests were two-tailed with significance set at *p* < 0.05. Given the exploratory nature of this study and the relatively small sample size, *p*-values are reported uncorrected for multiple comparisons; findings should be interpreted as hypothesis-generating rather than confirmatory. Effect sizes are provided alongside *p*-values to facilitate interpretation of the magnitude of observed effects. We emphasise that all significant associations reported herein require independent replication in larger cohorts before definitive conclusions can be drawn.

## 3. Results

### 3.1. Acute Behavioural and Acoustic Responses During Disbudding

**Figure 1:**
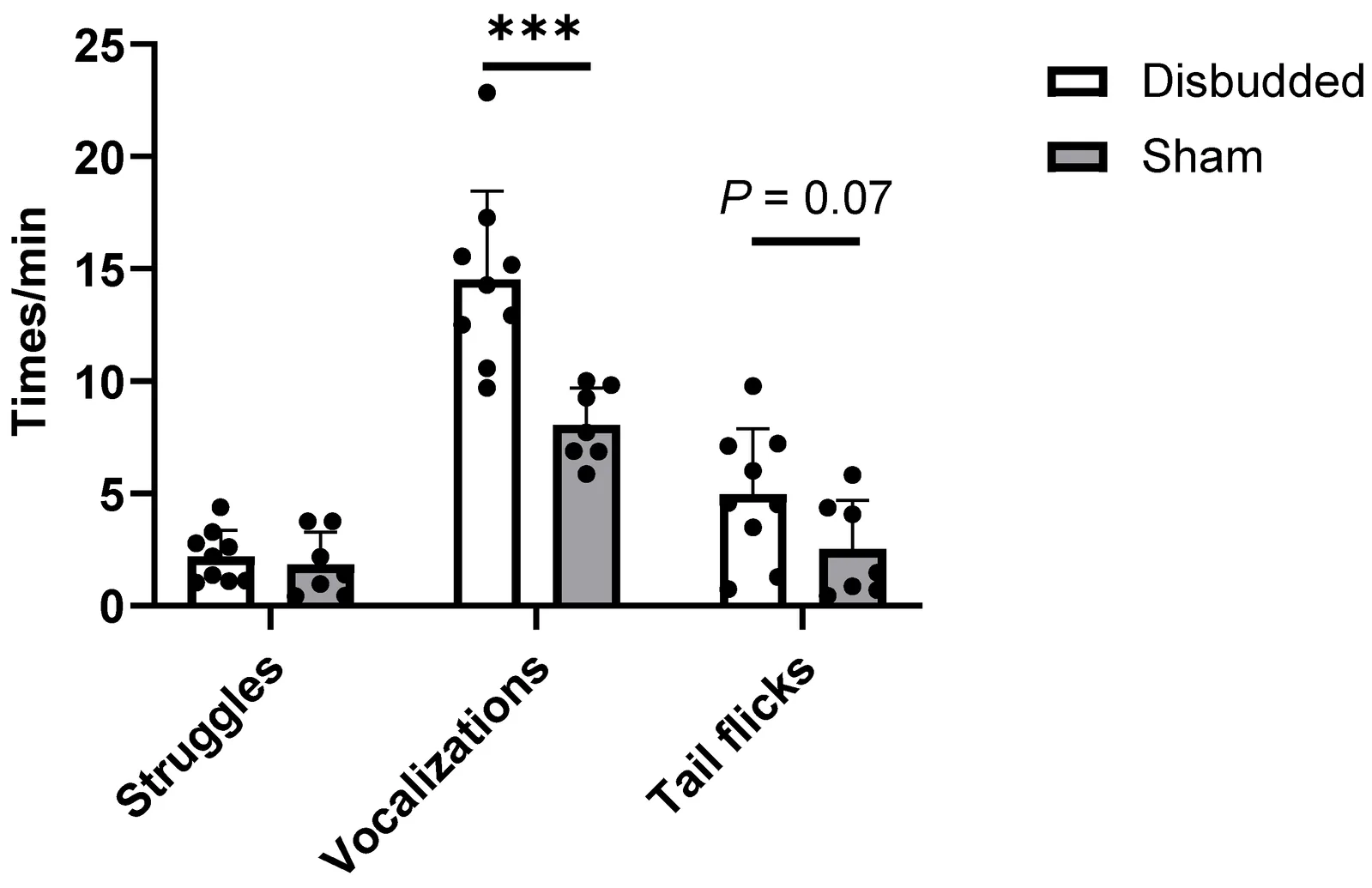
Frequencies of struggles, vocalisations, and tail flicks during the disbudding or sham procedure. Kids in the disbudded group (*n* = 9) underwent thermal cautery disbudding using a hot iron, and kids in the sham group (*n* = 7) were sham handled with an unheated iron. *** indicates significant difference between groups, and the indicated *p*-values represent trends toward significance. Data are presented as mean ± SD, with individual data points shown.

The frequencies of struggles, vocalisations, and tail flicks were compared between groups during the procedure using the Mann–Whitney *U* test (Figure 1). Disbudded kids vocalised significantly more frequently than the sham kids (disbudded: 14.54 ± 3.93 times/min, sham: 8.06 ± 1.64 times/min; *p* = 0.0007, effect size *r* = 0.8475). Tail flicks tended to be higher in the disbudded group compared to the sham group (disbudded: 4.97 ± 2.92 times/min, sham: 2.53 ± 2.17 times/min; *p* = 0.071, effect size *r* = 0.4511). However, there was no significant difference in the frequency of struggles between the two groups (disbudded: 2.20 ± 1.17 times/min, sham: 1.85 ± 1.43 times/min; *p* = 0.458, effect size *r* = 0.1854).

**Figure 2:**
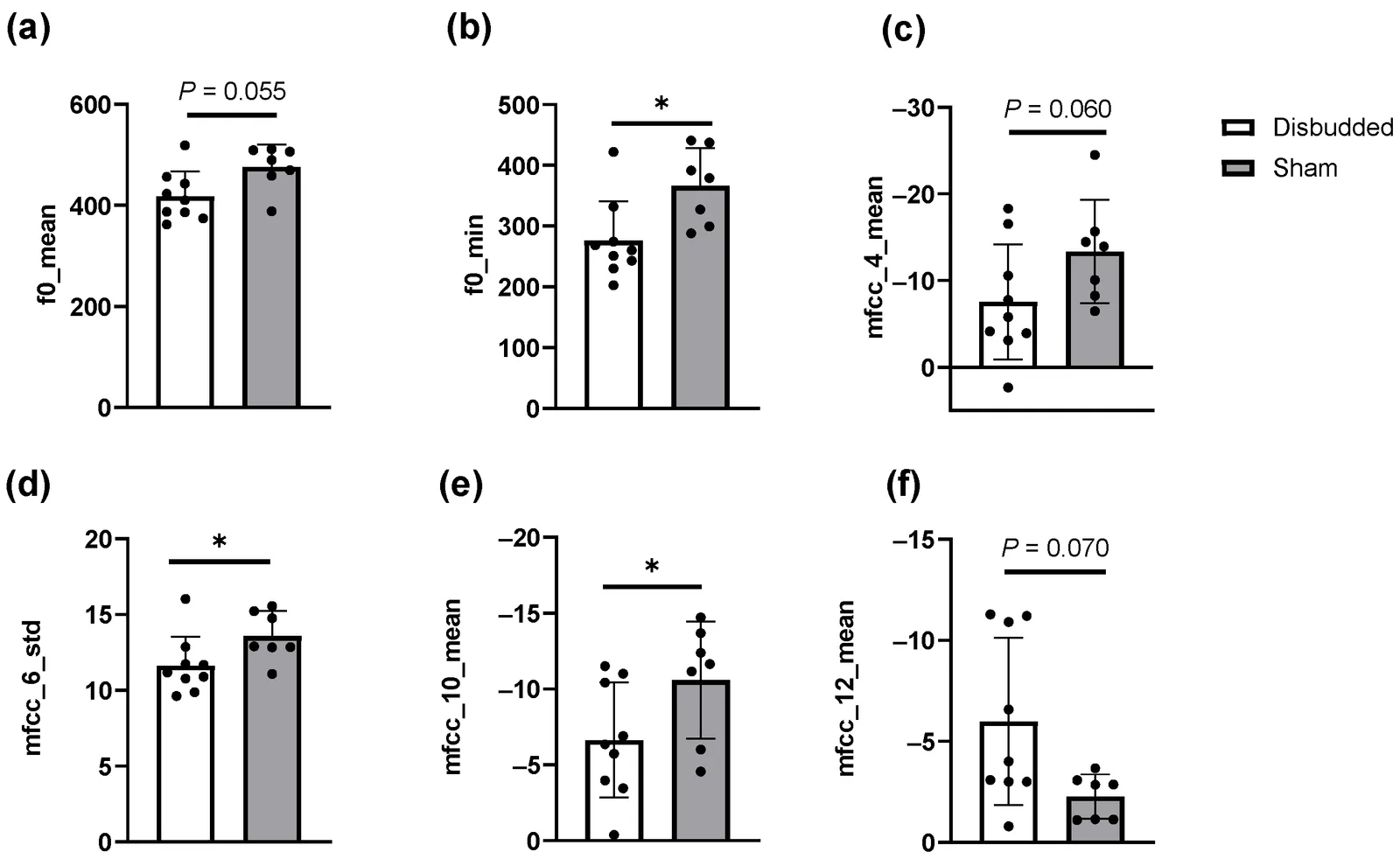
Mean acoustic features of vocalisations including (**a**) f0_mean, (**b**) f0_min, (**c**) mfcc_4_mean, (**d**) mfcc_6_std, (**e**) mfcc_10_mean, and (**f**) mfcc_12_mean during the disbudding or sham procedure. Kids in the disbudded group (*n* = 9) underwent thermal cautery disbudding using a hot iron, and kids in the sham group (*n* = 7) were sham handled with an unheated iron. * indicates significant differences between groups, and the indicated *p*-values represent trends toward significance. Data are presented as mean ± SD, with individual data points shown.

Acoustic analyses of the recorded vocalisations revealed that the mean fundamental frequency (f0_mean) did not significantly differ between the disbudded and sham groups (independent *t*-test, *p* = 0.056); however, it exhibited a large effect size (Cohen’s *d* = −1.02), indicating a biological trend (Figure 2a). In contrast, the minimum fundamental frequency (f0_min) was significantly lower in the disbudded group compared to the sham group (disbudded: 283.37 ± 65.68 Hz, sham: 366.13 ± 62.62 Hz; independent *t*-test, *p* = 0.020, Cohen’s *d* = −1.28; Figure 2b).

Significant alterations in vocal timbre were also observed. The value of mfcc_6_std was significantly lower in the disbudded group than in the sham group (Mann–Whitney *U*, *p* = 0.033, effect size *r* = −0.52; Figure 2d), and the mfcc_10_mean in the sham group was significantly higher than that in the disbudded group (independent *t*-test, *p* = 0.034, Cohen’s *d* = 1.15; Figure 2e). Additionally, there was a tendency for mfcc_4_mean to be higher in the disbudded group (independent *t*-test, *p* = 0.060, Cohen’s *d* = 1.00; Figure 2c), and mfcc_12_mean showed a trend toward being higher in the disbudded group compared to the sham group (Mann–Whitney *U*, *p* = 0.071, effect size *r* = −0.45; Figure 2f). Given these group-level acoustic differences, we next examined whether individual variation in these features within the disbudded group was associated with later phenotypic outcomes.

### 3.2. Body Weight Trajectories from 0 to 90 Days Post-Procedure

**Figure 3:**
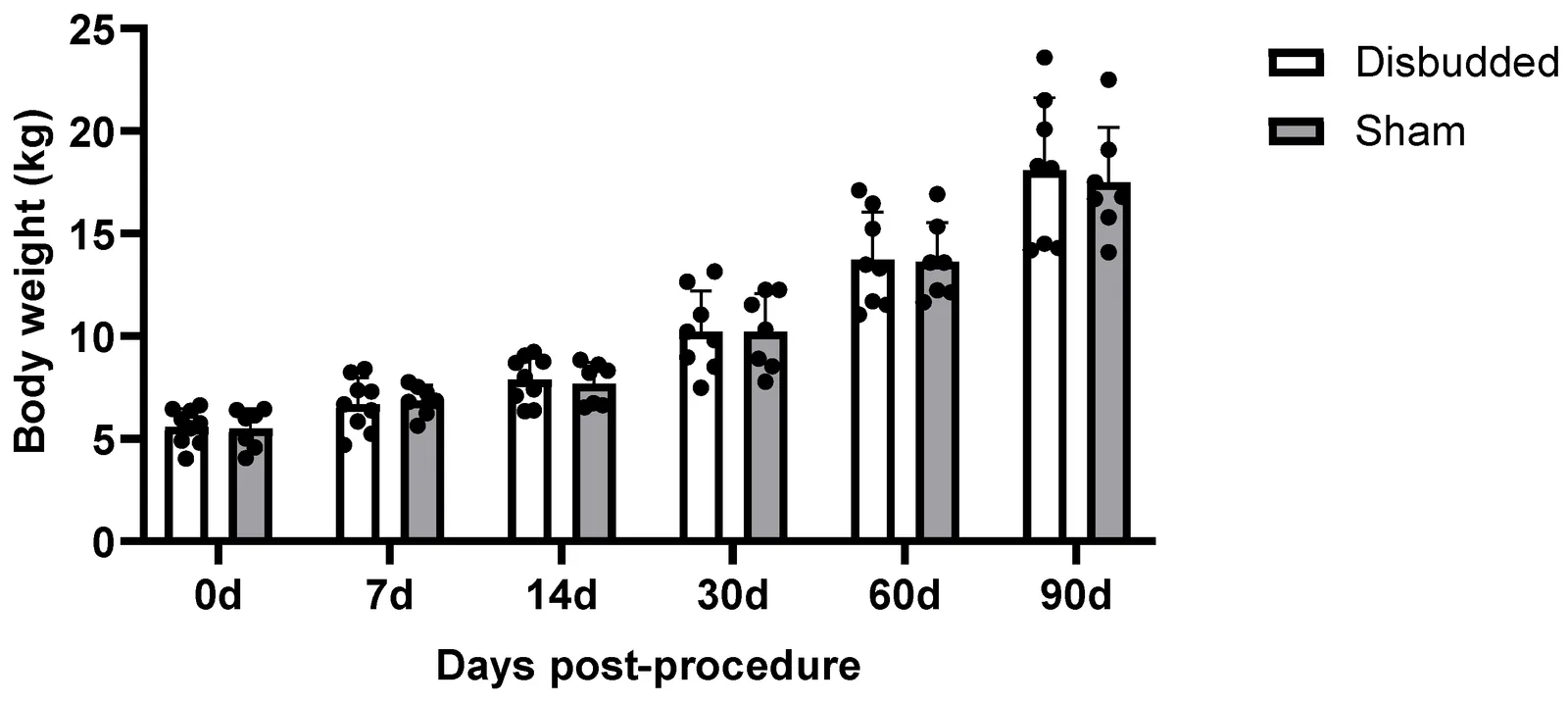
Body weight trajectories of disbudded (*n* = 9, reduced to *n* = 8 from day 30 onward due to one missing individual) and sham (*n* = 7) kids from the day of the procedure (day 0) to 90 days post-procedure. Data are mean ± SD. No significant differences were found between groups at any time point (all *p* > 0.05).

Body weight was measured on the day of the procedure (day 0) and at 7, 14, 30, 60, and 90 days post-procedure. At no time point did body weight differ significantly between the disbudded and sham groups (all *p* > 0.05; Figure 3). At 90 days, body weight was 18.09 ± 3.56 kg in the disbudded group and 17.50 ± 2.68 kg in the sham group (independent *t*-test, *p* = 0.727, Cohen’s *d* = 0.185; Figure 3). Body weight trajectories for both groups are shown in Figure 3.

### 3.3. Partial Correlations Between Vocal Features and 90-Day Post-Procedure Body Weight, Body Measurements, and Temperament Within the Disbudded Group

**Figure 4:**
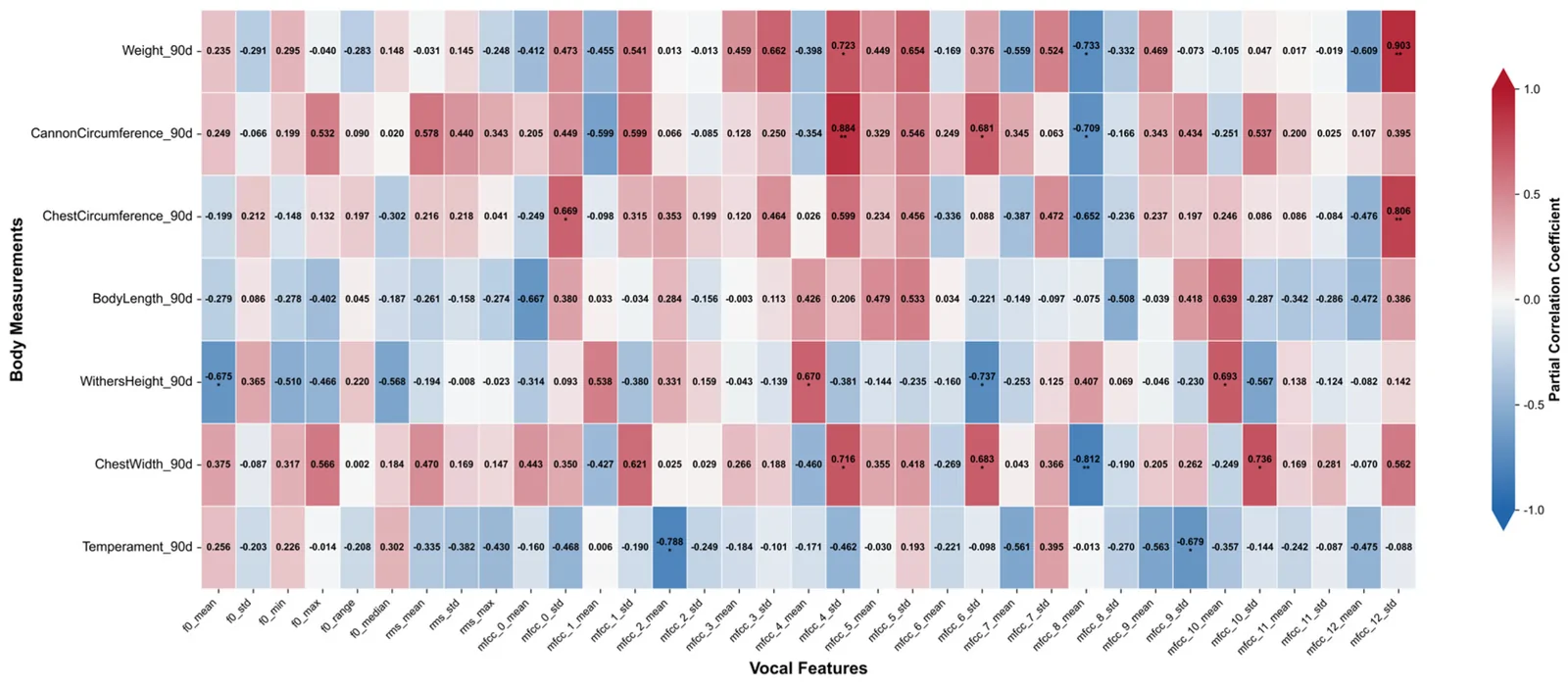
Partial correlation heatmap between vocal features during acute disbudding and body weight, body measurements, and temperament at 90 d post-procedure. Partial Spearman’s correlations were calculated within the disbudded group while controlling for body weight on the day of disbudding (*n* = 9). The colour gradient indicates the strength and direction of the partial correlation coefficients (red = positive, blue = negative), and the values within the cells represent the exact coefficients. Asterisks indicate significant correlations: * *p* < 0.05; ** *p* < 0.01.

Given the absence of group-level differences in body weight throughout the 0- to 90-day observation period, we examined whether individual variation in acoustic responses to disbudding was associated with subsequent growth within the disbudded group. The following partial correlations are exploratory and have not been adjusted for multiple testing. Partial correlation analyses were conducted within the disbudded group (*n* = 9) to examine the associations between the acute vocal features during disbudding and body weight, body measurements, and temperament at 90 days post-procedure while controlling for body weight on the day of the procedure (Figure 4). For 90-day body weight, mfcc_12_std was highly positively correlated (*r* = 0.903, *p* = 0.001), mfcc_4_std was positively correlated (*r* = 0.723, *p* = 0.028), and mfcc_8_mean was negatively correlated (*r* = −0.733, *p* = 0.025). Regarding temperament scores, mfcc_2_mean (*r* = −0.788, *p* = 0.012) and mfcc_9_std (*r* = −0.679, *p* = 0.044) showed significant negative correlations. In terms of body dimensions, mfcc_4_std was positively correlated with cannon circumference (*r* = 0.884, *p* = 0.002), while mfcc_12_std exhibited a positive correlation with chest circumference (*r* = 0.806, *p* = 0.009).

### 3.4. Representative Spectrograms, Post-Procedure Behaviour, Body Weight, and Their Correlation with Acoustic PC1 Score

**Figure 5:**
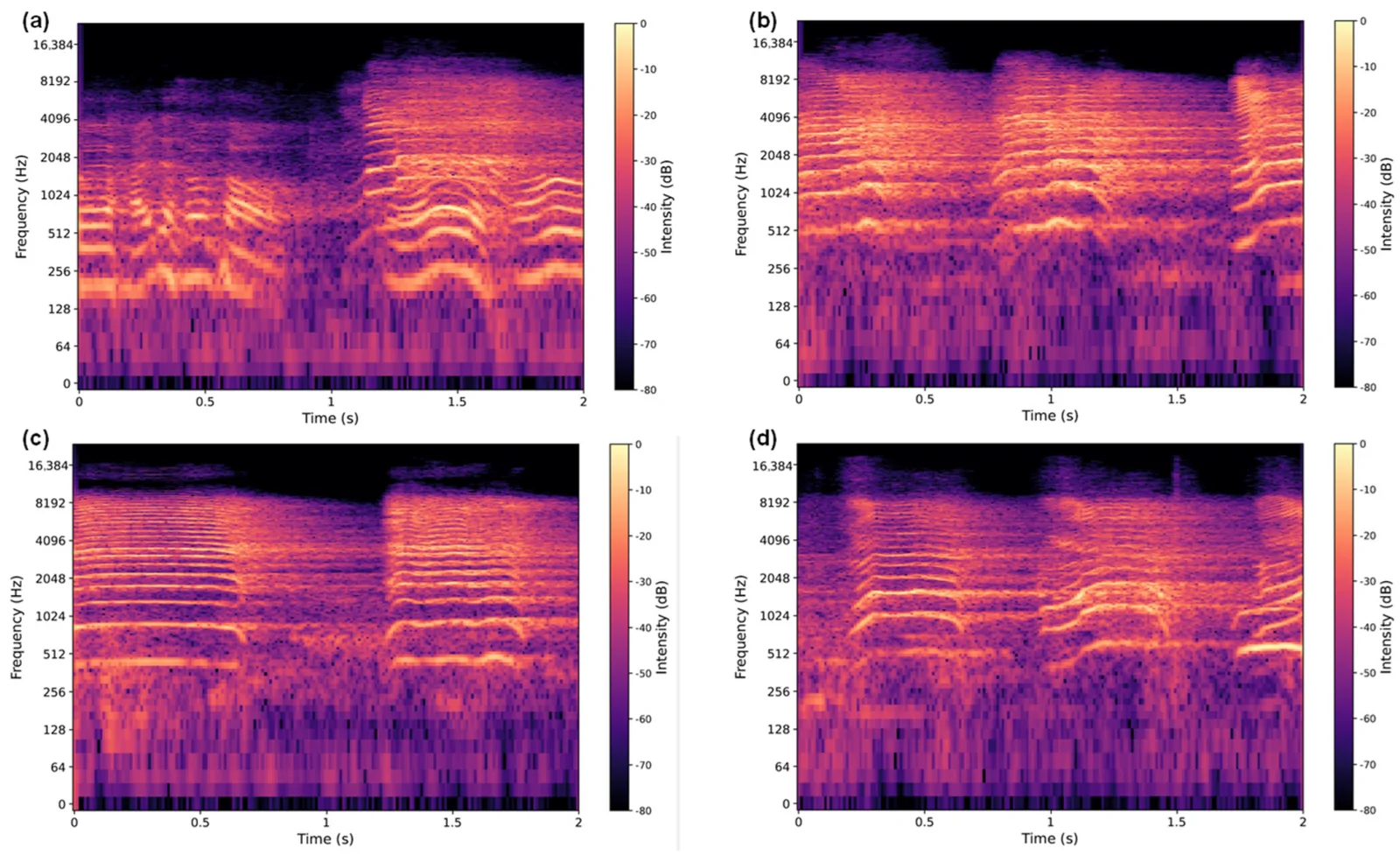
Representative spectrograms of vocalisations from subgroups with higher (H) and lower (L) acoustic PC1 scores within the disbudded and sham groups (see Section 2.7 for PCA-based classification): (**a**) Disbudded-H, (**b**) Sham-H, (**c**) Disbudded-L, and (**d**) Sham-L. The colour scale indicates acoustic energy intensity (dB). Vertical dark intervals between energy bands represent natural silent pauses between successive vocalisation pulses.

To generate a composite index within the disbudded group for subsequent analyses, PCA was performed on standardised acoustic features from the nine disbudded kids. PC1 explained 37.7% of the variance (loadings in Table A1). For descriptive comparison, PC1 explained 25.3% in the full dataset. Representative spectrograms of vocalisations from subgroups with higher (H) and lower (L) acoustic PC1 scores within the disbudded and sham groups are presented in Figure 5. In terms of overall energy distribution, the acoustic energy was primarily concentrated within the 0–4 kHz range across all samples, with negligible acoustic energy observed above 8 kHz. Furthermore, distinct intergroup differences were found in the fine time–frequency structures. Regarding spectral definition, the harmonic bands of the disbudded kids (particularly the representative individuals of the H subgroup) showed noticeable broadening, blurred boundaries, and diffuse characteristics, with high harmonics overlapping and lacking the sharp, coherent, and regular linear formant structures observed in the sham group. In the low-frequency energy distribution, the disbudded group (especially the H subgroup) exhibited more prominent and intensely coloured energy bands in the 128–256 Hz range, suggesting a concentration of energy at the lower fundamental frequencies. Furthermore, differences were observed in the temporal structure of vocalisations. Within a fixed analysis window, typical vocalisation impulses of the disbudded group generally exhibited longer single durations, appearing as two continuous energy bands, whereas the sham group typically showed three shorter and separated energy bands.

**Figure 6:**
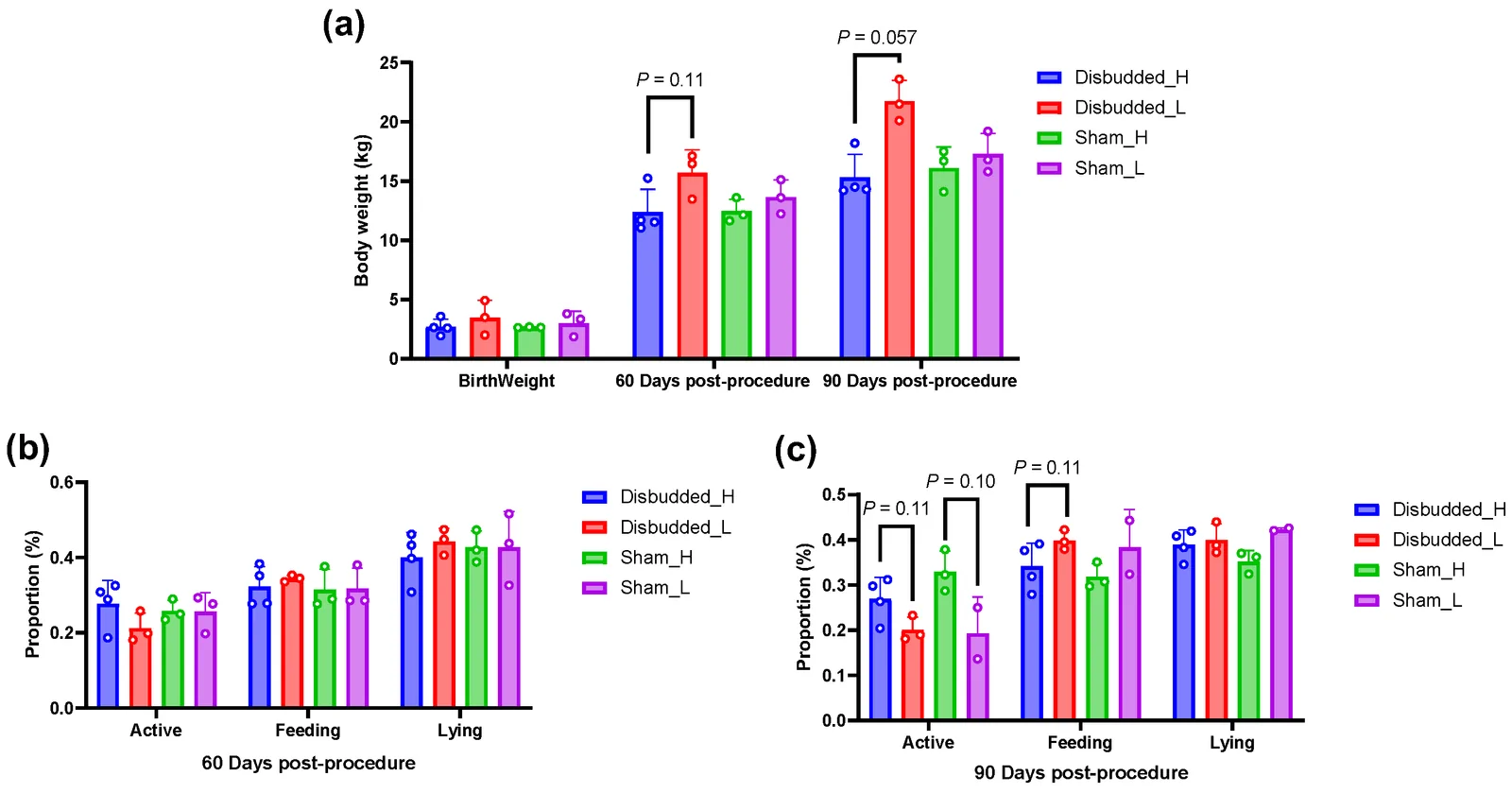
Body weight (**a**) and scan-sampling behavioural proportions (**b**) at 60 d and (**c**) at 90 d post-procedure. H and L denote subgroups with higher and lower acoustic PC1 scores, respectively, as derived from PCA clustering (see Section 2.7). *p*-values are provided for descriptive reference; subgroup comparisons are exploratory and not confirmatory due to small sample sizes. Data are presented as mean ± SD, with individual data points shown. PCA-based subgrouping included 13 kids (7 H: 4 disbudded, 3 sham; 6 L: 3 disbudded, 3 sham); the 90-day behavioural data exclude one L sham kid that escaped.

The four PC1-based subgroups (Disbudded-H, Disbudded-L, Sham-H, and Sham-L) were subsequently assessed for differences in post-procedure behaviour and body weight (Figure 6). At 60 days post-procedure, the Disbudded-L subgroup tended to have a higher body weight than the Disbudded-H subgroup (*p* = 0.11, effect size *r* = 0.597). By 90 days post-procedure, this trend persisted, with the Disbudded-L subgroup tending to have a higher body weight than the Disbudded-H subgroup (*p* = 0.057, effect size *r* = 0.719). In terms of behaviour, at 90 days post-procedure, the Disbudded-L subgroup showed a tendency toward a higher proportion of feeding behaviour compared with the Disbudded-H subgroup (*p* = 0.11, effect size *r* = 0.597), while the Disbudded-H subgroup exhibited a trend of a higher proportion of active behaviour compared with the Disbudded-L subgroup (*p* = 0.11, effect size *r* = 0.597). Lying behaviour proportions were similar among the four subgroups at both 60 and 90 days post-procedure, with no obvious trends identified.

Finally, the relationship between acoustic PC1 score and growth performance was further assessed using PC1 regression analyses. Within the disbudded group, acoustic PC1 was significantly and negatively correlated with body weight at 90 days post-procedure (Spearman’s *r* = −0.786, *p* = 0.036; linear *R*^2^ = 0.401). In contrast, no significant correlation was found between acoustic PC1 and body weight in the sham group (Spearman’s *r* = −0.257, *p* = 0.623; linear *R*^2^ = 0.000, interpreted cautiously given the exploratory nature and small sample size).

## 4. Discussion

This preliminary study indicates that acute vocal responses to disbudding not only differentiate painful from sham handling at the group level, but also may carry preliminary individual information associated with later growth beyond the immediate procedure, highlighting the potential of vocal phenotyping as a non-invasive tool for identifying at-risk kids in commercial settings.

Disbudded kids showed the expected pattern of elevated vocalisation and tail flick rates relative to shams, whereas struggling did not differ between groups despite comparable definitions to those used in previous studies [1,2]. The relatively low struggle frequencies in both groups suggest that, under the specific handling conditions of this study, restraint and handling did not elicit a sufficiently strong struggling response to discriminate between painful and non-painful treatments. This interpretation is supported by findings in piglet castration studies, where leg kicks and body flailing both discriminated castrated from sham castrated piglets [26]. However, the absence of a struggle effect in the present study, despite clear evidence of pain from vocal and acoustic measures, suggests that behavioural manifestation of pain may differ across species and procedures, and that struggling in goat kids may be less sensitive than in piglets under this specific handling context. Under conditions where handling itself is a potent stimulus, struggling may be a less specific indicator of disbudding pain than vocalisations or tail flicks.

Beyond behavioural counts, disbudding altered the acoustic structure of vocalisations. The spectral differences visualised in Figure 5—broadened, blurred harmonic bands and energy concentration at lower frequencies in disbudded kids—align with the MFCC shifts reported above and likely reflect pain-induced increases in laryngeal muscle tension and alterations in vocal tract configuration during phonation. Such biomechanical changes have been proposed as the basis for MFCC shifts in stressed vocalisations across several species [8,9]. MFCC-based features have also been used to classify stressed and non-stressed cattle vocalisations with high accuracy [27], and a recent machine learning study on goats identified MFCCs as key predictors for detecting physical distress [10]. The present study extends these cross-species findings to goat kids, demonstrating that acute disbudding pain produces specific and measurable changes in vocal timbre beyond call counts.

A key finding of this study was the observed association between acoustic features recorded during disbudding and body weight and temperament at 90 days post-procedure, after controlling for body weight on the day of the procedure. Given the exploratory nature of the analyses, this association should be interpreted as hypothesis-generating rather than as evidence of a predictive relationship. The dissociation of the PC1–growth correlation by treatment group—present in disbudded kids but absent in shams—raises the possibility that the relationship is specific to the pain context rather than reflecting a general acoustic–growth association. This pattern, together with the correlations between individual MFCC features and later phenotypic measures, is consistent with the hypothesis that vocal characteristics during an acute painful event may carry information about an individual’s underlying phenotype. One possible interpretation—though speculative—is that the acoustic features may be related to the intensity of the stress response, which could in turn be associated with energy allocation and growth. In goat kids, multimodal pain management during disbudding has been shown to partially attenuate acute stress responses [5], and pain mitigation strategies can affect subsequent health and performance outcomes [14]. More broadly, early-life painful procedures can have lasting phenotypic consequences in farm animals, including altered pain sensitivity [13] and growth [12,14]. However, evidence linking disbudding-induced pain directly to reduced weight gain is not uniform: one study in goat kids reported no detectable effect of cautery disbudding on weight gain over a 2-week window [28]. The present findings extend this literature by showing that, while group-level effects on growth may be inconsistent, individual differences in pain reactivity—as reflected in vocal responses—may offer hypothesis-generating insights for subsequent growth trajectories. Alternatively, vocal features may be partly related to stable individual traits—a ‘vocal phenotype’—that could be associated with both pain reactivity and later development. However, we emphasise that the present study was not designed to test causal mechanisms, and alternative explanations cannot be ruled out. The proposed ‘vocal phenotype’ should therefore be regarded as a preliminary hypothesis requiring further investigation, rather than a confirmed biological entity. However, the absence of the PC1–growth correlation in shams argues against a purely stable-trait interpretation; one possibility is that individual vocal differences are amplified specifically under high-arousal pain states, whereas under low-arousal conditions (as in sham handling) such differences may be less manifest. This interpretation is consistent with a cross-species study on ungulates showing that negative-valence contexts reduce the amount of individual information encoded in vocalisations, although certain acoustic parameters retain individual distinctiveness even across emotional states [17]. Thus, the two interpretations need not be mutually exclusive: a stable vocal phenotype may exist, but its association with phenotypic outcomes becomes detectable primarily when the pain context unmasks or magnifies individual differences in physiological reactivity. Individual vocal signatures have been identified in pigs, with acoustic features showing consistent within-individual patterns across stress conditions [29], and individual differences in goat kid vocalisations have been described, with sufficient interindividual variability to allow individual recognition [15,16]. The negative correlation between mfcc_2_mean and temperament score in the present study lends some support to the trait-based interpretation, although the temperament test used was a single brief scoring and should be interpreted cautiously. Regardless of the underlying mechanism, it should be emphasised that all correlation analyses in this study were exploratory, with *p*-values not corrected for multiple comparisons, and should therefore be regarded as hypothesis-generating rather than confirmatory. Independent replication in larger cohorts is essential before vocal features can be considered validated predictors of post-disbudding outcomes.

When kids were classified by acoustic PC1 into H and L subgroups, no significant differences in scan-sampled behaviours were found at 60- and 90-day post-procedure. This is consistent with previous reports [5], which also found no behavioural differences between treatment groups over the first 48 h after disbudding despite physiological effects. Although that window is considerably shorter than the 60- and 90-day assessments here, the consistent insensitivity of home-pen behaviours suggests they are less responsive to early pain than growth-related outcomes. The null subgroup findings are likely driven by the very small subgroup sizes (3–4 per group) and power loss from dichotomisation, rather than a true absence of effect. Critically, the continuous analysis revealed a treatment-specific relationship that was lost upon categorisation. This dissociation underscores the value of continuous over categorical analysis and suggests that the vocal growth link is robust despite the absence of subgroup differences in behavioural time budgets. Future studies should adopt continuous analytical approaches or ensure larger sample sizes.

Several limitations warrant acknowledgement. The correlational analyses presented in this study are exploratory and hypothesis-generating. No adjustments for multiple testing were applied, as the primary aim was to identify candidate acoustic markers for future hypothesis-driven investigations rather than to confirm predefined predictions. Only female kids were used because male kids are rarely disbudded in commercial practice, as horn size has been associated with semen quality in goats [18], and the findings cannot be generalised to males. No analgesic or local anaesthetic was administered, consistent with the prevailing commercial practice in the region and with several published disbudding studies [1,2]. This design choice, however, limits direct comparability with studies conducted under pain control. Various pain mitigation protocols have been investigated, including local nerve blocks and multimodal strategies [4,30], but none have yet achieved complete attenuation of pain responses. Studies in piglets have shown that local anaesthesia with lidocaine reduces acute vocal and cortisol responses during castration [31]. However, at least one study reported no detectable effects of this intervention on subsequent weight gain [31], suggesting that the biological pathways linking early pain to later performance may not be simply alleviated by short-term analgesia. Importantly, the broader literature has noted that little is known about the effects of pain mitigation on longer-term health and performance outcomes in disbudded goat kids [14], highlighting the relevance of the present exploratory findings. Taken together, the present findings identify vocal acoustics as a candidate early marker of vulnerability at 90 days post-procedure, but the exploratory nature of the correlational analyses and the modest sample size warrant cautious interpretation and independent replication. Furthermore, a repeated-measures approach was not applied to the longitudinal body weight data; separate tests were performed at each time point, which may have increased the risk of Type I error and did not account for individual variation in growth trajectories. These findings should therefore be regarded as hypothesis-generating and require independent validation in larger cohorts before any definitive conclusions can be drawn.

Several alternative explanations for the observed associations should also be considered. The correlations reported in this study may be influenced by genetic differences among individuals, maternal effects, inherent physiological characteristics, or environmental factors that were not controlled for. For example, individual variation in stress reactivity may have pre-existing genetic or maternal components that influence both vocal structure and later growth. Based on the current study design, it is not possible to determine which of these mechanisms, if any, underlies the observed relationships. Future studies with larger sample sizes and controlled breeding or longitudinal designs are needed to disentangle these possibilities.

## 5. Conclusions

In conclusion, thermal disbudding without analgesia altered both the rate and acoustic structure of goat kid vocalisations. Acoustic PC1 was associated with later body weight in disbudded but not sham handled kids, which may indicate that vocal features indicative of pain reactivity may be related to either acute stress reactivity or stable individual vocal phenotypes that become manifest under pain challenge. These findings identify vocal acoustics as candidate early markers that warrant further investigation and highlight their potential utility for non-invasive monitoring of post-disbudding vulnerability, provided that they are validated in independent, larger-scale studies.

## Figures and Tables

**Table 1:** Ethogram of behaviours recorded during the disbudding or sham procedure.

Behaviour	Definition	Reference
Vocalisation	Each distinct audible bleat, characterised by an opening and closing of the mouth.	adapted from [20]
Tail flick	A vigorous vertical tail wagging in any direction from its resting position and back.	[20]
Struggle	A bout of slight or vigorous movements of the hind legs and/or trunk, or an attempt to escape from restraint.	[1]

**Table 2:** Content of Trillat’s five-point temperament test.

Score	Behaviour
1	Calm, no movement
2	Calm with occasional movements
3	Calm with some more movements but without shaking the scale
4	Abrupt episodic movements without shaking the scale
5	Permanent episodic movements and shaking the scale

**Table 3:** Ethogram for scan-sampling of behaviour at 60 and 90 days after the procedure.

Behaviour	Definition
Lying	The kid is recumbent with its sternum and all four legs in contact with the ground.
Feeding	The kid is standing with its head lowered into the feed trough.
Active	All other behaviours not captured by ‘Lying’ or ‘Feeding’, including standing, walking, running, playing, and social interactions.

## Data Availability

The data that support the findings of this study are available from the corresponding author upon reasonable request.

## Abbreviations

The following abbreviations are used in this manuscript:

F0	Fundamental frequency
MFCCs	Mel-frequency cepstral coefficients
PCA	Principal component analysis
PC1	First principal component
RMS	Root mean square
pYIN	Probabilistic YIN algorithm
NSAIDs	Nonsteroidal anti-inflammatory drugs

## Appendix A

**Table A1:** Principal component loadings for acoustic features in the disbudded group.

Feature	PC1_Loading	PC2_Loading
mfcc_4_mean	0.355	−0.015
mfcc_12_mean	0.353	0.217
mfcc_3_mean	−0.344	0.155
f0_mean	−0.335	0.159
loudness	0.296	−0.074
mfcc_7_mean	0.263	0.264
mfcc_5_mean	−0.259	0.364
mfcc_1_mean	0.248	−0.167
mfcc_8_mean	0.224	0.053
f0_min	−0.221	−0.384
mfcc_0_mean	0.215	−0.186
mfcc_2_mean	0.178	0.235
mfcc_10_mean	0.167	0.343
mfcc_11_mean	0.166	−0.212
mfcc_9_mean	0.033	0.315
mfcc_6_mean	−0.019	0.408

Note: PC1 accounted for 37.7% of the total variance; PC2 accounted for 24.5%.
